# The Influence of Physical Factors on Kelp and Sea Urchin Distribution in Previously and Still Grazed Areas in the NE Atlantic

**DOI:** 10.1371/journal.pone.0100222

**Published:** 2014-06-20

**Authors:** Eli Rinde, Hartvig Christie, Camilla W. Fagerli, Trine Bekkby, Hege Gundersen, Kjell Magnus Norderhaug, Dag Ø. Hjermann

**Affiliations:** 1 Section for Marine Biology, Norwegian Institute for Water Research, Oslo, Norway; 2 Department of Biosciences, University of Oslo, Oslo, Norway; 3 Natural History Museum, University of Oslo, Oslo, Norway; University of Aveiro, Portugal

## Abstract

The spatial distribution of kelp (*Laminaria hyperborea*) and sea urchins (*Strongylocentrotus droebachiensis*) in the NE Atlantic are highly related to physical factors and to temporal changes in temperature. On a large scale, we identified borders for kelp recovery and sea urchin persistence along the north-south gradient. Sea urchin persistence was also related to the coast-ocean gradient. The southern border corresponds to summer temperatures exceeding about 10°C, a threshold value known to be critical for sea urchin recruitment and development. The outer border along the coast-ocean gradient is related to temperature, wave exposure and salinity. On a finer scale, kelp recovery occurs mainly at ridges in outer, wave exposed, saline and warm areas whereas sea urchins still dominate in inner, shallow and cold areas, particularly in areas with optimal current speed for sea urchin foraging. In contrast to other studies in Europe, we here show a positive influence of climate change to presence of a long-lived climax canopy-forming kelp. The extent of the coast-ocean gradient varies within the study area, and is especially wide in the southern part where the presence of islands and skerries increases the area of the shallow coastal zone. This creates a large area with intermediate physical conditions for the two species and a mosaic of kelp and sea urchin dominated patches. The statistical models (GAM and BRT) show high performance and indicate recovery of kelp in 45–60% of the study area. The study shows the value of combining a traditional (GAM) and a more complex (BRT) modeling approach to gain insight into complex spatial patterns of species or habitats. The results, methods and approaches are of general ecological relevance regardless of ecosystems and species, although they are particularly relevant for understanding and exploring the corresponding changes between algae and grazers in different coastal areas.

## Introduction

Kelp forest and sea urchin dominated barrens are considered to be two alternative stable states of an ecosystem [1], [2] and the presence of the two states is mutually exclusive. Due to reinforcing feedback mechanisms both states have a high degree of resilience; the kelp forest inhabits sea urchin predators that may prevent overgrazing, whereas the barren areas lack suitable habitats for sea urchin predators [2]. The transition from sea urchin dominated barrens to kelp seems to occur when sea urchin density decrease below critical thresholds [2]–[4]. The focus of this study is how the distribution of the kelp *Laminaria hyperborea* and the sea urchin *Strongylocentrotus droebachiensis* is affected by physical factors.

After decades of destructive grazing and barren ground formation, sea urchin populations decline and kelp forests recover along parts of the Norwegian coast in the NE Atlantic. Since the 1970s, the sea urchin *Strongylocentrotus droebachiensis* (O.F. Müller) has occurred in high densities in sheltered or moderately wave exposed areas between 63°N and 71°N [5]–[8]. From the early 1990s, a gradual decline in sea urchin density and a subsequent recovery of kelp (*L. hyperborea* and *Saccharina latissima*) has been reported in the southern part of this area, and by 2007 the southern border of the barren ground had shifted northward to 65.5°N [9]. The observed recovery of kelp is not uniform, and a mosaic with remaining barren grounds within the reforested coastline (see [9]) indicates a complex interaction of factors determining kelp recovery when sea urchin density decreases. Both natural mortality incidents [10]–[13] and field experiments [4], [14], [15] have shown that kelp forests recover when sea urchin density decline. However, the succession pattern toward a recovered “climax kelp forest” will vary according to the distance to the nearest kelp forest [4] and with local processes such as dispersal capabilities, competition, recruitment and grazing pressure [16], [17]. Obviously, physical factors at different scale levels, that influence kelp growth, will be important for kelp recovery.

The underlying mechanisms for the reduced sea urchin densities and the northward shift in the southern border for barren grounds are not understood. It may be linked to increased temperature caused by global warming and to increased predation pressure on different life stages of the sea urchins [9]. A recent study shows low sea urchin recruitment within the recovery area at 65.7°N in 2008–2010 [18]. This has been linked to increasing incidents of maximum temperatures above 10°C [18] and increased predation pressure from the edible crab (*Cancer pagurus*) moving northwards [19]. The study showed high recruitment in colder water north of the recovery area (70.7°N), that could indicate a large scale influence of temperature with latitude. However, observations of high densities of small-sized sea urchins (2–4 cm, i.e. 2–3 years old according to [19]) at the same latitude in 2012 (H. Christie, unpublished data) indicate local differences in sea urchin recruitment success, uncoupled to temperature. Hence reduced densities of sea urchins and the resulting recovery of kelp may be an effect of large scale changes in both temperature (across latitude or longitude) and predator distribution and density. Moreover, it may involve mechanisms that operate on a more local scale creating patches were sea urchins are able to maintain high densities in e.g. habitat refuges not accessible for predators [20].

Important physical factors for presence, growth and development of kelp and sea urchins varies with latitude and longitude at different scales, and can be used for predicting the distribution of kelp and sea urchin dominated barren grounds [9], [21]. Light is indirectly important for sea urchins through its influence on algae growth and hence food availability. Temperature and light vary with latitude and depth whereas salinity and wave exposure vary from the ocean to the coastal areas along the longitudinal gradient. Salinity declines from full marine conditions in the outer oceanic areas to variable and brackish conditions in the surface of inner fjords, where sea urchins may face salinities below their tolerance limit [22]. Wave exposure also declines from the offshore areas to the sheltered coastal archipelago and fjords.

*Laminaria hyperborea* dominates rocky substrate in shallow areas above critical light depth (approx. 30 m) with moderate and high wave exposure [21], [23], and intact *L*. *hyperborea* forests have persisted in the most wave exposed areas ever since the grazing event was first recorded [6]. *L. hyperborea* seems to have optimal growth conditions in mid-Norway (62–65°N) due to the combination of light irradiance, day length and temperature [24]. The Atlantic current brings warm water northwards in offshore areas that border and mix with colder water in the coastal current inshore of the Norwegian Sea [25]. Thus there is variation in the sea temperature along the coast-ocean gradient that might influence kelp growth and sea urchin survival. Local differences in current speed [23] and terrain characteristics, such as depth, curvature and slope (the two latter being proxies for substrate; steep/rugged areas implying rocky substrate and flat areas in valleys implying soft sediments), also affects kelp plants growth conditions and thereby their distribution [21], [26], [27].

Field investigations of the distribution of kelp and sea urchins in the affected area in the last decade (2004–2011) provide the opportunity to analyze and identify physical factors that may explain the spatial distribution of the two species. Hence, in this study we wanted to investigate how well physical factors can explain the observed distribution of the two species, and to identify the most important of these factors. This was done by means of statistical analysis of presence-absence data of the kelp *Laminaria hyperborea* and of the sea urchin *Strongylocentrotus droebachiensis* from field investigations in this period, relating the observed distributions to a number of relevant physical factors. By focusing on areas where kelp have been reported to be grazed by sea urchins [6], [8], [9], we postulated that presence of kelp and lack of sea urchins implies recovery of kelp, and that presence of sea urchins implies persistence of barren grounds or macrophyte- (kelp or other macrophytes) covered areas still grazed by sea urchins. Available temperature data for the period 1990–2007 [28] were analyzed to reveal if large scale variation in temperature across the north-south and the coast-ocean gradient could be linked to the observed patterns of kelp recovery. We also wanted to assess the extent of kelp recovery and sea urchin persistence by using the statistical models to predict the spatial distribution of the two alternative states, and to determine how the patterns of recovery varied between regions.

## Material and Methods

No specific permissions were required for the field studies in any of the visited locations. Most studies were performed on assignment from Norwegian nature management authorities. The field studies did not involve endangered or protected species.

### The study area and field sampling

**Figure 1:**
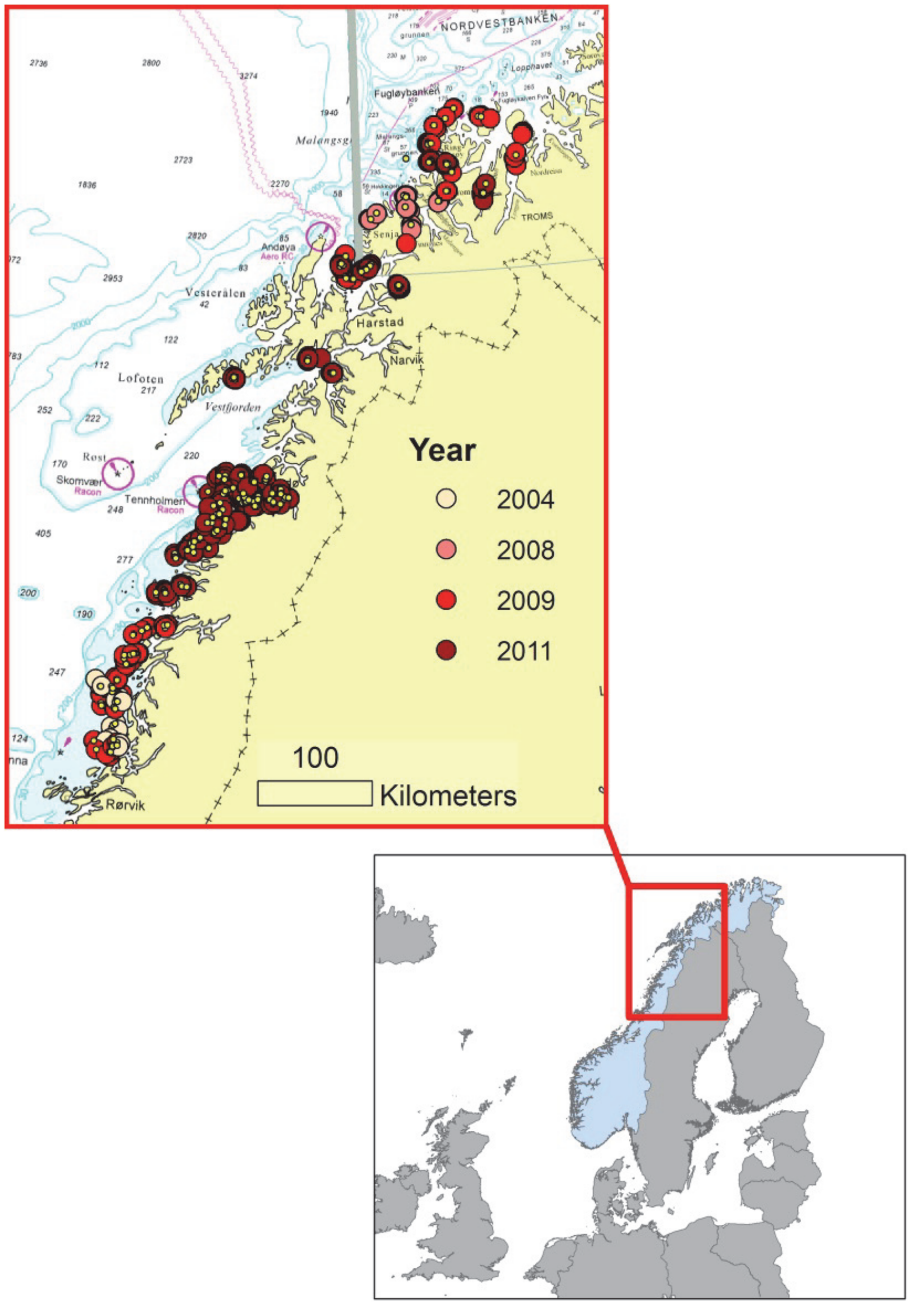
The study area. Map of the study area along the north Norwegian coast 65°–68°N, within the northeast Atlantic, coded for sampling year. Large points (n = 1220) are model data for the statistical analyses, whereas small yellow points (n = 403) are test data for evaluation of the models.

Data were compiled from field investigations carried out in a number of projects in the period October 2004 to June 2011 in previously grazed areas (65°–68°N, **Figure 1**) along the Norwegian coast during summer and late autumn. Using underwater cameras with depth sensors or by scuba diving we recorded presence (i.e. scattered to high density) and absence (the species not observed) of the kelp *Laminaria hyperborea* and of the sea urchin *Strongylocentrotus droebachiensis*. The majority of sites (75%, examined by underwater camera) were selected stratified and randomized within chosen study areas to achieve a representative overview of the status of the two species. The chosen study area was delineated by a circle in GIS. Selectable pixels within the study area was delimited to the depth interval 0–40 m, and 30–50 pixels (25×25 m) per study area were selected at random within each area. The size of the observed sites within each pixel was about 1×1 m. The remaining sites were sampled along transects (underwater camera or scuba diving) of various length and approx. 1–2 m wide) from shallow to deeper water (<40 m). Sites from the most wave exposed areas, where sea urchin barren grounds have never been observed (cf the simplified wave exposure classes in [29]), were excluded from the analysis in order to focus on the processes involved within the previously grazed area. When predicting kelp recovery and sea urchin persistence we further restricted the study area to encompass only areas where *L. hyperborea* kelp forests were assumed to have been grazed, i.e. areas shallower than 30 m depth and moderately exposed to waves [29]. These restrictions resulted in a prediction area of 1 561 km^2^ (i.e. approx. 2.5 mill. pixels at the chosen spatial resolution of 25×25 m). Additionally, to reduce the problem with spatial autocorrelation among data sampled along transects, we removed sites closer than 25 m from another site. The data filtering resulted in 1 623 available recordings of presence/absence of kelp and sea urchins within the study area (**Figure 1**). If there were several observations at the same site, we used the most recent observation. The proportion of recovered kelp (i.e. presence of kelp and absence of sea urchins) and presence of sea urchins (with and without kelp) among the sampled sites were 20% and 16% respectively. Only 0.2% of kelp forest sites (i.e. sites with common to high densities of kelp) had presence of sea urchins, and 64% of the sites had none of the species.

### Physical factors (modeled predictor variables)

11 predictor variables (satisfactory uncorrelated with Spearmans correlation rho <0.63) were applied; including depth, slope, terrain curvature, optimal light index, wave exposure index, current speed (minimum and maximum values), temperature (mean), salinity (maximum), latitude and a coast-ocean gradient (for details, cf **Table S1**). The coast-ocean gradient was included instead of longitude, due to the northeast orientation of the coastline and a high correlation between latitude and longitude, and to reveal the importance of this gradient in explaining large scale distribution patterns. Temperature, wave exposure, salinity, and current speed also varies along this gradient, and were included in the analyses since their co-variance with the gradient was not too large (rho = −0.24, −0.53, −0.51 and 0.62, respectively). Several environmental factors, such as temperature and day length, vary with latitude, and might affect kelp and sea urchins. As the available temperature data at the most detailed spatial resolution (800 m) are from one summer month (August 2009, **Table S1**), we included latitude and the coast-ocean gradient to encompass other aspects of the variation in temperature along these gradients than variation in summer temperature.

All variables were available as GIS layers (description in **Table S1**); current speed, temperature and salinity at a spatial resolution of 800 m, the others at a spatial resolution of 25 m. The 800 m predictors were resampled to 25×25 m resolution to allow for prediction at a more detailed scale. All field samples and predictor variables were integrated in ArcGIS 10.0. For the statistical analyses we used field-measured depth rather than GIS-modeled depth.

### Statistical analyses and evaluation of predictive models

The relationship between physical factors and the presence and absence of *Laminaria hyperborea* recovery and *Strongylocentrotus droebachiensis* persistence was analyzed using General Additive Models (GAMs) and Boosted Regression Trees (BRTs). GAM is a flexible method that can identify and characterize non-linear relationships. Instead of fitting functions by simple least squares, each function in GAM is fitted using a scatterplot smoother (a cubic spline or a kernel smoother) and an algorithm that simultaneously estimates all included functions [30]. BRT, on the other hand, combines the strengths of regression trees and boosting, by gradually increasing emphasis on observations modelled poorly by the existing collection of trees. Each added tree predicts the residuals from the previous tree to improve the predictive performance. BRTs are able to model interactions and sharp discontinuities in species' responses to environmental gradients, they automatically select variables, and are robust to outliers and missing data [30]. We applied GAM in order to detect general, large scale relationships between species and environmental factors [31] and BRT in order to discover important interactions of predictors [32], [33]. BRT models have also been reported to perform better than GAMs in predicting species distribution [32], [34]. The models were developed using the programming language R, version 2.15.0 [35], using the packages *mgcv* [36] and *Dismo* [37] for the GAM and BRT analyses, respectively.

In the GAMs we used cubic regression spline as the penalized smoothing basis, as this is a low-order spline that creates a smoothing between the joints that are not visible to the human eye [30]. To avoid overfitting, the dimension of the basis used to represent the smooth term (k) was set to 3 for single predictors and to 6 for interactions. The R-package *MuMIn* [38] was used for model selection of GAM-models, providing AICc values (the model selection criteria) and a ranked selection table for all possible combinations of variables (i.e. candidate models). We also analyzed the influence of some specific interactions of interest, i.e. the interactions between depth and wave exposure and between depth and latitude. Candidate models with ΔAICc<4 were regarded as receiving a similar degree of support from the data and were included in an average model for each species, as recommended by [39]. The relative importance of the predictors in the GAMs was calculated in *MuMIn* as the sum of the Akaike weights over all models including the predictor among the subset of models with ΔAICc<4 [38]. We examined the residuals for the best GAMs for normality, independency and constant variance, and did not detect any violations of these assumptions.

In the BRT modeling approach [33] we used tree complexity (tc) equal to 5 (the number of splits in each tree, also called the interaction depth), and bag fraction equal to 0.5 (the default setting of the fraction of the training set observations randomly selected to propose the next tree in the expansion, and suggested by [33]). The AUC value for the BRT kelp model increased from 0.92 (tc = 3 and 4) to 0.93 for tc = 5. Hence we chose to use tc = 5 for both species. The BRT analysis provides a ranked list of the relative contribution from each predictor. The measures are based on the number of times a variable is selected for splitting, weighted by the squared improvement to the model as a result of each split, and averaged over all trees [40]. The relative influence of each variable is normalized to sum to 100, with higher numbers indicating stronger influence on the response.

The data set was randomly divided into a training set (75%, i.e. 1 220 registrations) and a test set for model evaluation (25%, i.e. 403 registrations). These data sets were used for both kelp and sea urchin modeling. The R package *PresenceAbsence* [41] was used to evaluate the BRT and GAM models when applied to the test data (AUC values of the ROC curves and calibration plots). Moran's I of the residuals of the best models was calculated in ArcGIS to check for spatial autocorrelation.

### Status of kelp recovery and sea urchin persistence at large and regional scale

By applying the best predictive models for *Laminaria hyperborea* recovery and *Strongylocentrotus droebachiensis* persistence to previously grazed areas within the study area (i.e. moderately wave exposed areas between 65 and 68°N shown in **Figure 1**) we have created maps of kelp recovery and areas with continued occurrence of sea urchins for the period 2004–2011. To reveal how the distribution of these two states of the kelp forest (*sensu* [42]) varied between regions along the coast, we divided the study area into five equally sized latitudinal regions (each region covers a latitudinal and longitudinal range of 424 and 121 km respectively; region 1 the most southern and region 5 the most northern). *Dismo* [37] was used to make prediction maps. We estimated the size of the recovered kelp area and the areas with continued persistence of sea urchins for the full range of cut-off probabilities to assess the appropriate condition (species occurrence or absence). The remaining area (i.e. total area minus the area of the two states) was defined to be without the two species. We also applied the Youden index, also called the true skill statistic, as criteria for selecting the optimal cut-off value (i.e. the optimal threshold criteria called “Max sens + spec” in [41]). This index identifies the threshold that maximizes sum of sensitivity and specificity and thereby optimizes the possibility to differentiate between presence and absence of a condition when equal weight is given to sensitivity and specificity. To estimate the differences in recovery of kelp and persistence of sea urchins between regions across the latitudinal gradient, we have applied the Youden index (cf above) of the models. We did not have sufficient data for a post-hoc validation of the predictions. However, the models are evaluated by the test data, as described above.

### Analysis of climate change in the period 1990–2007

Temperature data [28] with a course spatial horizontal resolution (1/2 degree longitude times 1/3 degree latitude, i.e. 23×38 km at 65.5°N) were analyzed to reveal if any large scale variation in temperature across latitude and along the coast-ocean gradient could be linked to the observed patterns of kelp recovery. The data set consisted of interpolated temperatures for the four quarters January-March, April-June, July-September, and October-December for each of the years 1990–2007. The data have a vertical resolution of 28 standard hydrographic depth levels from 0 to 500 m. We selected temperature estimates from 63.6°N (south of the southern border of our study area) to 70.3°N (equal to the northern border of our study area), from the 10 m depth level (in total 5 964 points). To assess the relationship between these large scale temperature data and the predictors latitude, the coast-ocean gradient, season (quarter) and year, we extracted the values to the data points and applied mixed GAM (using package *mgcv*) with temperature as the response variable. Season was included by treating month as a cyclic factor. We accounted for temporal correlation by assuming an autoregressive model of order 1.

## Results

### Presence of recovered *Laminaria* hyperborea kelp

**Figure 2:**
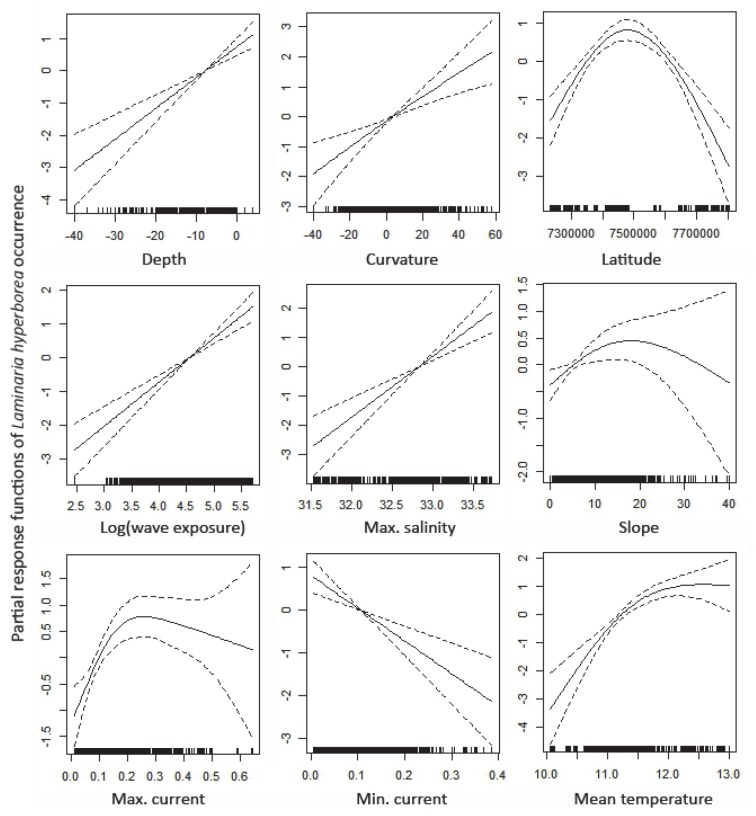
GAM for kelp recovery. The partial response plots of the best GAM (lowest AICc) for presence of the kelp *Laminaria hyperborea*.

The best candidate GAM for describing presence of recovered *Laminaria hyperborea* (i.e. presence of *L. hyperborea* kelp and absence of the sea urchin *Strongylocentrotus droebachiensis*) included all predictors but the coast-ocean gradient and the optimal light index, and explained a high amount of the deviance, i.e. 37.1%. Cut-off values between 0.2 and 0.3 resulted in 79 to 84% of the test data being correctly classified. The partial response plots of the predictors in this model are shown in **Figure 2**. Eight models received significant support from the observations (i.e. ΔAICc<4, see [39], **Table S2**). The Relative Importance (RI) of the predictor variables in this subset of models equals 1 (i.e. they are included in all of the eight models) for all predictors except depth (RI = 0.50), wave exposure (RI = 0.50), the depth-wave exposure interaction (RI = 0.50), the coast-ocean gradient (RI = 0.30) and the optimal light index (RI = 0.28). The interaction between depth and latitude did not achieve enough support to be included as a predictor in any of the models in the subset of best models.

Listed according to their relative importance in the BRT analysis (below), the partial response plots of the best GAM imply that *L. hyperborea* mainly recover at:

- *high degree of wave exposure* - the probability for recovery increased with increased degree of wave exposure, probably due to improved growth conditions.
- *saline areas* - the probability of recovery increased with increased salinity. This may be related to the coupling of higher salinity in wave-exposed, outer areas.
- *ridges* - the probability of recovery increased from negative to positive curvature values, i.e. as the terrain change from basin to shoals. This may be related to better light conditions and to the presence of appropriate substrate (hard bottom).
- *medium high summer temperatures* - the probability of recovery increased with increasing mean temperature in August until approx. 12°C.
- *medium high latitudes* - the probability for recovery peak at medium high latitudes (∼67°N) within the study area.
- *shallow water* - the probability for recovery increased from deep to shallow areas, probably due to improved light conditions.
- *medium strong currents* - the probability of recovery increased with high maximum currents up to a certain threshold level where it flattened off. The probability of recovery was reduced when maximum and minimum current speed was high. This response may be connected to a positive effect of current speed on kelp growth, but too strong currents can cause problems with detachment and settlement.
- *medium slopes* - the probability of recovery increased with increasing slope until about 10–15 degrees. This is probably related to the likelihood of appropriate substrate (rock), and problems with attachment at steeper slopes.

**Figure 3:**
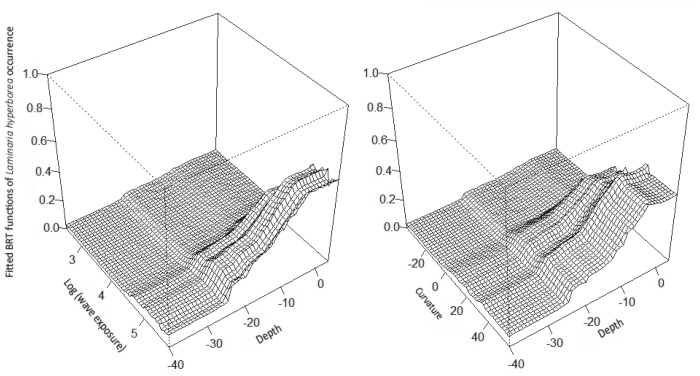
Interactions in the BRT model for kelp recovery. Predictions based on the main interactions from the BRT model of kelp *Laminaria hyperborea* recovery: wave exposure x depth (left, interaction size  = 48.9) and curvature x depth (right, interaction size  = 40.9).

The resulting BRT model explained a larger amount of the deviance (65%) than the best GAM. Cut-off values between 0.2 and 0.3 resulted in 86 to 90% of the test data correctly classified. The partial response plots of the BRT model (**Figure S1**) showed similar responses for kelp recovery as the GAM to the predictors depth, slope, curvature, wave exposure, temperature and salinity. However, BRT showed a more threshold-like response to e.g., temperature, and a more complex response to latitude and current speed. The main interactions included in the BRT model were between wave exposure and depth, and between curvature and depth (**Figure 3**). These interactions imply an increased probability of recovery in areas that are both shallow and wave exposed. Similarly, there was an increase in probability of recovery in shallow, hilly areas. According to the BRT model, the five most important predictors for recovery of *L. hyperborea* were, in decreasing order: wave exposure, maximum salinity, curvature, temperature, and latitude (**Figure S1**).

To summarize the results described above; recovery of the kelp *Laminaria hyperborea* mainly occurred at shallow ridges in wave exposed, saline and warm areas at lower latitudes.

The evaluated models (the best GAM, the average GAM with and without interactions, and the BRT model) all received high AUC-values (≥0.9) when applied to the test data (i.e. excellent models according to the classification of [43]. Also, the calibration plots indicated that all of the four tested kelp *Laminaria hyperborea* models performed very well (**Figure S2**). Based on AUC-values, the average GAMs and the best GAM were equally good (AUC = 0.9). Based on this and the principle of parsimony, the most parsimonious model was the best GAM, which did not include interactions. However, the BRT model outcompete the GAMs in the evaluation (AUC = 0.93). Moran's I (−0.06) did not indicate spatial autocorrelation in the residuals of the BRT model (p = 0.2).

### Presence of Strongylocentrotus droebachiensis

**Figure 4:**
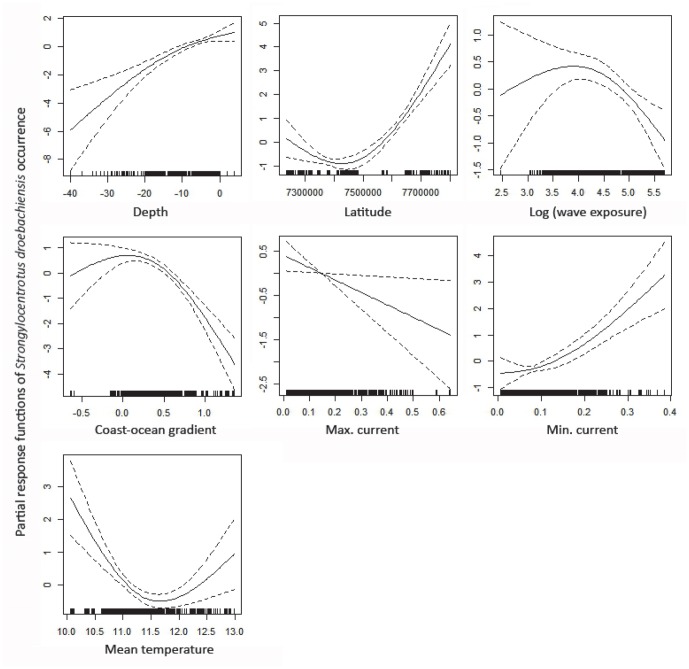
GAM for sea urchin persistence. The partial response plots of the best GAM without interactions for presence of sea urchin *Strongylocentrotus droebachiensis*.

The best GAM (based on AICc and the principle of parsimony, cf below) for describing the sea urchin distribution included all predictors but slope, curvature, optimal light index and salinity, and explained a relatively high amount of the deviance, i.e. 31.4%. Cut-off values between 0.2 and 0.3 resulted in 80 to 84% of the test data correctly classified. The partial response plots of this model are shown in **Figure 4**. The analysis provided 36 competing models with ΔAICc < 4 (**Table S3**). As for kelp recovery, the interaction between depth and latitude did not achieve enough support from the data to be included among the best sea urchin GAMs. Latitude, the coast-ocean gradient, minimum current speed and mean temperature was included in all of the models (i.e. relative importance, RI, of the predictors is equal to 1). RI of the other predictors was in decreasing order; the interaction between depth and wave exposure (0.83), maximum current speed (0.79), maximum salinity (0.55), optimal light index (0.43), slope (0.43), curvature (0.29), depth (0.17) and wave exposure (0.17). The best of these models (lowest AICc) included the interaction between depth and wave exposure, and excluded curvature, slope and the optimal light index.

Listed according to their relative importance in the BRT analysis, the partial response plots of the best GAM model imply that *Strongylocentrotus droebachiensis* mainly persist at:

- *high latitudes* - the probability of *S. droebachiensis* persistence increased from approx. 67.5°N and further north. This southern limit corresponds to a ca. 260 km northwards displacement of the barren ground limit from 2007 [9].
- *shallow water* - the probability of *S. droebachiensis* persistence increased from deep to shallow areas. This could be due to a greater supply of food through higher algae production in these areas.
- *low mean summer temperature* - the probability of *S. droebachiensis* persistence was high at low mean summer temperatures, and decreased with increasing values. For higher temperatures than approx. 12°C the response was vaguer.
- *intermediate position along the coast-ocean gradient* - the probability of *S. droebachiensis* persistence was at maximum at the intermediate part of the coast-ocean gradient, and decreased further out and further in along this gradient. The decrease in the outer area was rather vague with a wide confidence envelope due to few data.
- *low maximum and high minimum current speed* - the probability of *S. droebachiensis* persistence decreased with increasing maximum current speed and increased with minimum current speed. Hence high maximum current speed was less favorable for sea urchins, but they seemed to be positively influenced by having at least some current speed.
- *low wave exposure* - the probability of *S. droebachiensis* persistence decreased with increasing wave exposure. The response was more unclear for low exposures with a wide confidence envelope.

Unlike the *Laminaria hyperborea* GAM model, the best GAM for explaining the distribution pattern of *Strongylocentrotus droebachiensis* included the coast-ocean gradient and excluded curvature and maximum salinity. Hence curvature at 525 m resolution seemed to have little influence on sea urchins, whereas some variation along the coast-ocean gradient seemed to be important for sea urchin occurrence. Salinity is obviously an important factor for sea urchins, but the modeled maximum values in August 2009, at the sampled sites, did not contribute sufficient information to be included in the best sea urchin GAM. To summarize the results, sea urchins seem to persist in shallow, relatively sheltered, cold areas at high latitudes. Unlike kelp recovery, some factor(s) associated to the coast-ocean gradient, is important for the sea urchins.

**Figure 5:**
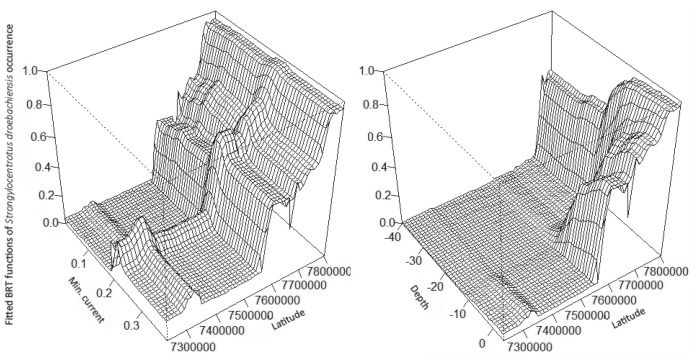
Interactions in the BRT model for sea urchin persistence. Predictions based on the main interactions from the BRT model of occurrence/persistence of sea urchin *Strongylocentrotus droebachiensis*: depth x latitude (left, interaction size  = 205.5) and minimum current speed x latitude (right, interaction size  = 91.2).

Based on the 11 predictors, the established BRT model for sea urchin *Strongylocentrotus droebachiensis* persistence explained 78% of the deviance in the data. Cut-off values between 0.2 and 0.3 resulted in 87 to 89% of the test data correctly classified. The response plots of the BRT model (**Figure S3**) showed similar responses as the most parsimonious GAM to the predictors; depth, latitude and maximum current speed. However, the response to wave exposure, temperature, minimum current speed and to the coast-ocean gradient differed from the GAM. The response to wave exposure was a small decrease with increasing exposure in the BRT model. The response to mean summer temperature was a distinct decreasing trend with increasing temperatures. For minimum current speed there seemed to be an optimal value equal to about 0.2 m/s for sea urchin persistence. The response to the coast-ocean gradient implied higher probability of sea urchins towards inner areas than the response curve in the GAM implied. The main interactions in the BRT model for sea urchins were between depth and latitude and between minimum current speed and latitude (**Figure 5**). These interactions implied increased probability of sea urchins in shallow areas at high latitude compared to the southern areas and in areas with minimum current speed equal to about 0.2 m/s. South of 68.4°N, the BRT model implied no variation in the persistence of sea urchins with depth or latitude. However, north of 68.4°N, the probability of sea urchin occurrence in shallow waters increased with latitude. The interaction between minimum current speed and latitude showed a similar pattern. South of 68.4°N, there is no influence of latitude, but north of this border the probability of sea urchin occurrence increase with latitude. The signal of an optimal current speed of about 0.2 m/s for sea urchin persistence applies for the entire latitudinal gradient. The five most important predictors to the distribution of *S. droebachiensis* was in accordance to the BRT-model in decreasing order; latitude, depth, temperature, the coast-ocean gradient and minimum current speed (**Figure S3**).

The evaluated sea urchin models (the best and the average GAM with and without interaction, and the BRT model) all received relatively high AUC-values (>0.8) when applied to the test data. Also, the calibration plots indicated that each of the four tested models for sea urchin persistence performed well (**Figure S4**).Based on AUC the evaluated GAMs were equally good (AUC = 0.8). Hence applying the principle of parsimony, the best GAM is the simple model without interactions. Similar to kelp recovery, the BRT model outcompete the GAMs by an AUC-value of 0.9. Moran's I (−0.04) did not indicate spatial autocorrelation in the residuals of the BRT model (p = 0.4).

### Sea urchin persistence and kelp recovery at large and regional scale

**Figure 6:**
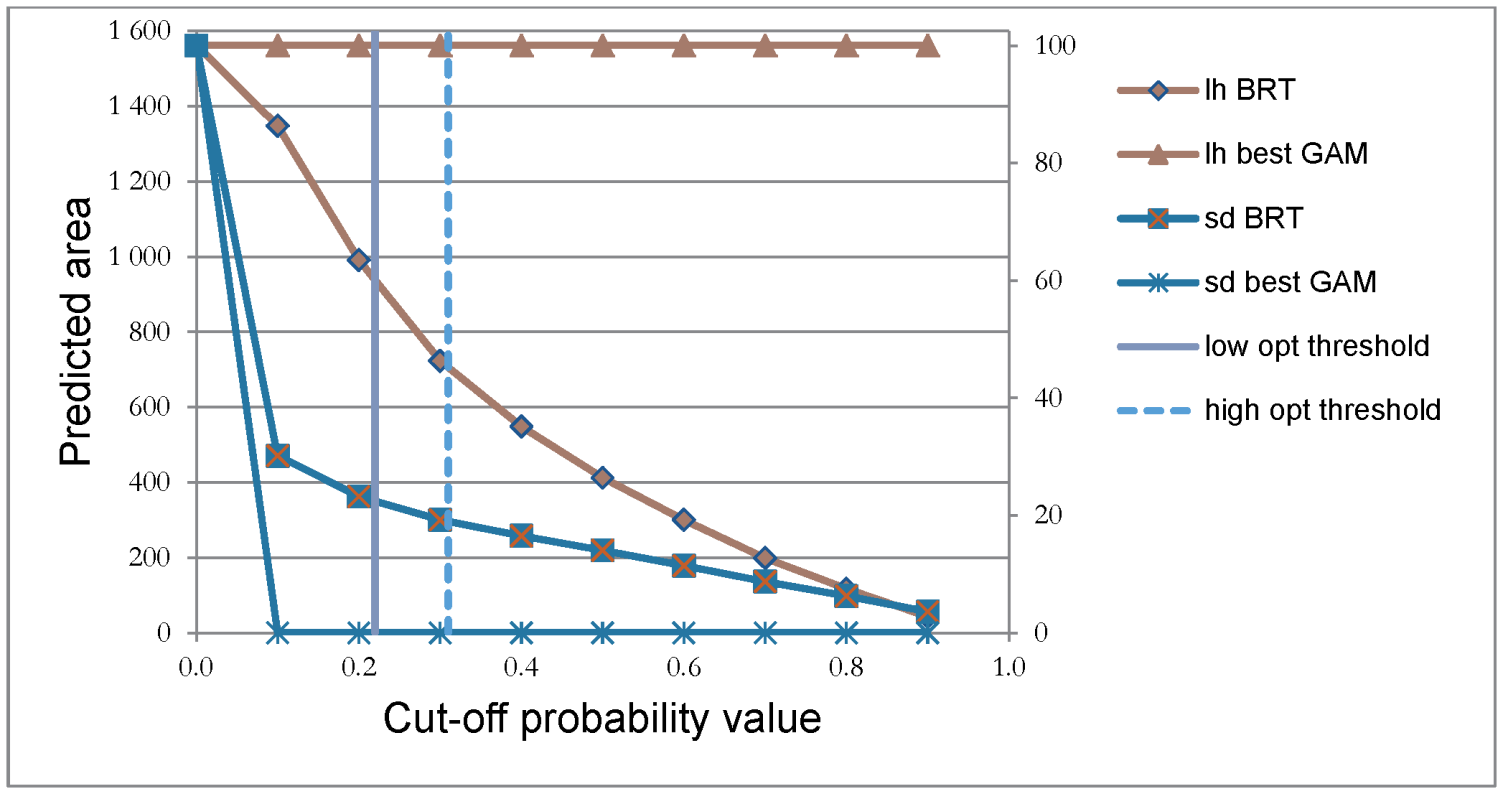
The extent of kelp recovery within the study area. Size of the predicted area (in km^2^ on the left axis and in percent of the study area on the right axis) modeled to be areas with recovery of kelp (*Laminaria hyperborea*, lh) and with persistence of sea urchins (*Strongylocentrotus droebachiensis*, sd), given different cut-off probabilities for the best GAMs and for the BRT models. The low and high optimal threshold lines show the lowest and highest cut-off value according to the Youden index for the best models.

**Figure 7:**
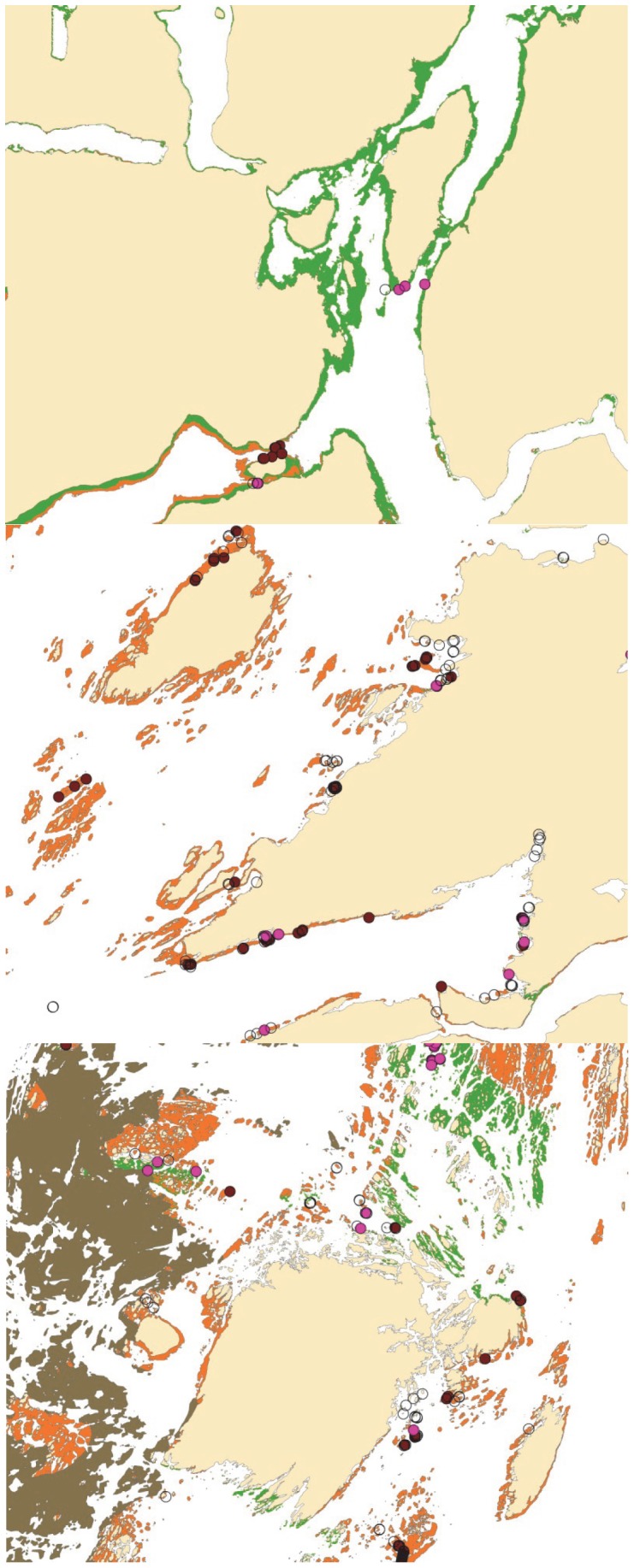
Maps showing predicted kelp recovery and sea urchin persistence. Predicted recovery of kelp *Laminaria hyperborea* (orange) and persistence of sea urchin *Strongylocentrotus droebachiensis* (green) based on the BRT models in region 1 (65°N, lower panel), region 3 (67°N, middle panel) and region 5 (69°N, upper panel). The map further shows land (beige), wave exposed areas where kelp never has been grazed (leather brown), field observed presences of *L. hyperborera* (brown circles), *S. droebachiensis* (violet circles) and absences of both species (open circles). White areas include shallow areas with predicted absences of the two species, areas deeper than 30 meters, as well as sheltered areas that are excluded from the predicted area. Scale 1∶100 000.

When applied to the study area, the best GAM for kelp recovery implied that the whole study area has a high probability of kelp recovery irrespective of cut-off probabilities (**Figure 6**). Similarly, the best GAM for sea urchin persistence implied that the probability of sea urchin presence is very low in the study area. The BRT models for kelp recovery and sea urchin persistence gave a more nuanced pattern (**Figure 6**), and the size of the area defined to have kelp recovery or sea urchin persistence depended on the selected cut-off probability. Examples of the predictions for three regions (region 1 in the south, region 3 in the middle, and region 5 in the north), based on the BRT-models, are shown in **Figure 7**. Given cut-off probability values between 0.2 and 0.3, suggested by the Youden index for the best GAM and BRT models, the BRT models claims that there has been a recovery of kelp within about 45–60% of the study area (700–940 km^2^), that sea urchins persists in about 20% of the area (312 km^2^), and that 20–35% of the area (312–546 km^2^) most likely lack both of the modeled species. Such areas could be experiencing a succession phase and be vegetated by other macroalgae species.

The splitting of the model area into five equally sized regions across latitude, displayed a marked difference in the area distribution of sea urchins presence between the three southern regions and the two most northern regions (**Table 1**). About 0.1 to 4% of the shallow coastal area in the three southern regions was still dominated by sea urchins, whereas sea urchins persisted in 30–70% of these areas in the two northernmost regions. The difference across regions with respect to recovery of *L. hyperborea* was less marked, with recovery of 55 to 75% in the southern regions and 38% and 19% in the two northernmost regions (**Table 1**) and with a peak of recovery in region 2.

**Table 1:** Estimates of recovered kelp area within 5 regions.

	Reg 1	Reg 2	Reg 3	Reg 4	Reg 5
Recovery of Lh	186.7 (56%)	246.9 (75%)	194.1 (55%)	114.7 (38%)	46.7 (19%)
Persistence of Sd	5.5 (2%)	0.4 (0.1%)	15.2 (4%)	94.5 (31%)	179.7 (73%)
Regrowth other macroalgae	141.7 (42%)	79.8 (24%)	140.7 (40%)	96.5 (32%)	18.5 (8%)
Total area	333.9	327.1	350.1	305.7	244.9

Predicted area (in km^2^) of kelp (*Laminaria hyperborea*, Lh) recovery and sea urchin (*Strongylocentrotus droebachiensis*, Sd) persistence in each of five regions along the Norwegian coast, where region 1 is the southernmost region. Areas with regrowth of other macroalgae were estimated as 100% minus the area with kelp recovery and the area with sea urchin persistence. We used the cut-off probability values equal to the Youden index (YI) for the BRT model for kelp recovery (0.27) and sea urchin persistence (0.31). The numbers in brackets are the size of the area in percentage of the total study area.

### Climate changes in the period 1990–2007

The mixed GAM analysis of the temperature data showed a significant temperature decrease northwards to about 68.5°N, and a slight increase further north (**Figure S5**). It also showed a temporal change in temperature with a warm period in the early 90s and around 2005, a significant influence of season (cold winters and warm autumns), and a significant influence of the coast-ocean gradient with coldest temperature in intermediate areas along the coast-ocean gradient (**Figure S5**). P-values for approximate significance of smooth terms were less than 0.001 for all of the included predictors. The model explained a high degree of the variation in the data (R^2^_adj_ = 0.79).

**Figure 8:**
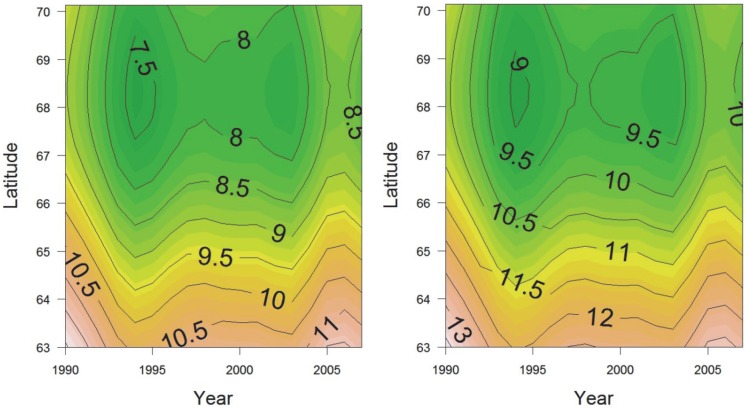
Summer sea water temperature along latitude in the period 1990 to 2007. Modeled sea water temperature at 10(left) and July (right) at latitudes between 63–70°N in the period 1990 to 2007, at an intermediate position along the coast-ocean gradient. The analysis is based on data from the Temperature Atlas developed by [28].

The latitudinal variation in temperature in the end of June in the period 1990–2007 (**Figure 8**) indicates that water warmer than 10°C reached northwards to about 66°N in the two warm years 1990 and 2005. Further, it implies that the area south of 64°N have experienced temperature above the critical limit for sea urchin larvae (i.e. 10°C) in this month, each year from 1990 to 2007. From about 2004 a similar pulse of warm water as in the early 90s occurred, reaching almost the same northern latitude (67.5°N). One month later (the end of July), the critical temperature level reached north to about 67°N in all years (**Figure 8**).

## Discussion

The study shows that physical factors cause some general large scale and small scale variation in recovery of kelp *Laminaria hyperborea* and persistence of the sea urchin *Strongylocentrotus droebachiensis* in a previously grazed area in the NE Atlantic. The large scale trends occur along the north-south and the coast-ocean gradient, and can both be related to temperature, explaining the observed borders for kelp recovery along these gradients. The large scale trend along the coast-ocean gradient may also be linked to wave exposure and salinity. Interactions between physical factors (i.e. wave exposure/depth and curvature/depth for kelp and depth/latitude and current speed/latitude for the sea urchins), as revealed by the BRT analysis, are needed to explain the observed mosaic patchiness of sea urchins within areas experiencing kelp recovery. These interactions may represent a direct physical influence on sea urchin and kelp growth and development, but they might also constitute a substitute for unknown important interactions. The achieved knowledge on the influence of the physical factors to kelp recovery and the established distribution models may be used to predict where kelp recovery is likely to occur in other grazed areas, and may be used to design research and monitoring programs of sea urchin-kelp dynamics.

The study underlines the usefulness of GAM and BRT models in ecological studies, and the advantage of applying several models to elucidate both general trends and more complicated interactions between involved factors. The gain of using a combination of methods is also recently recommended by [44] (using GAM and Maxent). BRT combines insights and techniques from both statistical and machine learning traditions [33] and has a great potential in developing models on species and habitat distribution as a function of environmental variables, but is so far rarely used in ecological studies [45].

The recovery of kelp implies reduced sea urchin densities [10]–[13]. Hence, the observed recovery in the south and the persistence of sea urchins in the north (which is also shown by [9]) indicates the influence of factor(s) that varies along the latitudinal gradient. [18] suggests that high temperatures may cause sea urchin recruitment failure in the south. Analysis of the Temperature Atlas data in combination with the analysis of the distribution of sea urchins and kelp supports the conclusion that the large-scale variation northward may be attributed to climate change. The decrease in sea urchin density within the southern part (<67°N) of the overgrazed area in the early 90s occurred simultaneously with a particularly warm period for this area (**Figure 8** **)**. A second, warm period influenced the southern part of the study area in the years 2004–2007. This latter period coincides with the period when recovery of kelp was reported to reach 65.5°N [9]. The warm water may have caused reduced densities of the cold water sea urchin species and allowed kelp recovery in these areas. [46] found that the critical upper limit for normal development of *Strongylocentrotus droebachiensis* larvae was 10°C. In 2004–2007, the sea water temperature at 10 m depth in the end of June was above 10°C south of 65°N (**Figure 8**). This is above the critical temperature limit for larvae development, and during an important time period for sea urchin larval development [47], [48]. The coldest water temperature (<8°C) in the end of June for all years in the period 1990–2007 was recorded north of 67.5°N. At this latitude, the border for kelp recovery was recorded in 2011, which might be caused by the cold water north of this border, enhancing sea urchin recruitment and development. This assumption is strengthened by the finding of high recruitment of *S. droebachiensis* in this region [18]. [49] and [50] found optimal gonad development and growth of *S. droebachiensis* at 10°C and reduced fitness at higher temperatures. Hence, in addition to recruitment failure due to reduced sea urchin larvae development, the increased temperature levels south of 67.5°N are likely to reduce the production of sea urchin larvae as well as adult sea urchins performance due to reduced fitness (i.e. reduced growth and survival).

The GAM analysis indicates maximum probability of kelp recovery at intermediate latitudes, but the corresponding response plot in the BRT analysis showed no clear pattern with latitude. This is in contrasts to the response plots for the sea urchins that showed a clear and uniform latitudinal pattern (increased probability of sea urchins from about 67.5°N and northward) using both GAM and BRT. These differences in response may be due to the time lag from reduced sea urchin density and regrowth of kelp, through variations in the succession process. Succession speed and succession pattern might vary due to differences in local physical factors and due to biological interactions, e.g. competition between algae species, and grazing by the red sea urchin *Echinus esculentus*, which seems to be favored by declining green sea urchin densities [9], [51]. These factors are likely to differ from those responsible for reducing sea urchin density. Hence recovery of kelp south of the recently established northern border for recovery may not follow the same pattern with latitude as the sea urchins. The reduced probability of kelp recovery and high probability of sea urchin persistence north of 67.5°N found in the GAM analysis strengthens the observation of this area as the new northern border for recovery of kelp.

The recovery of kelp and the persistence of sea urchins also vary along the coast-ocean gradient, and the distribution of the two species are affected in opposite manner by factors that vary along this gradient. The most influential of these factors are wave exposure and salinity. The GAM and BRT analysis showed an increased probability for recovery of kelp with increasing levels of wave exposure and salinity. This influence could be related to improved growth conditions for kelp. Increased wave exposure within the range of the study area are likely to have positive influence on kelp growth, through moving algal fronds and hence maximizing the area available to trap light, as well as maintaining a high nutrient flux [52]. A positive influence of wave exposure on *Laminaria hyperborea* growth was shown by e.g. [23], [53], [54]. The influence of salinity on kelp recovery may capture a link between higher salinity levels in wave-exposed, outer areas, which provide excellent growth conditions for kelp compared to more brackish water in sheltered coastal areas (cf. [23], [53] and references therein). The salinity at the inner limit for *L. hyperborea* is assumed to be between 25 and 30 psu [23]. Additionally, areas with high wave exposure and high salinity are also close to areas that were never affected by grazing. This will increase the supply of kelp spores to nearby areas and improve the possibility for rapid kelp recovery when sea urchin density is reduced. Salinity is an important factor for sea urchins [55], although the maximum salinity in summer had low importance in the sea urchin model at the spatial scale at which this information was available (800 m). Minimum salinity in spring at a finer resolution would perhaps improve the sea urchin models.

The coast-ocean gradient had a relatively high influence in the BRT-model for *Strongylocentrotus droebachiensis*, as the fourth important factor, whereas it was less important (the ninth most important variable) for the corresponding model for kelp recovery. The probability of *S. droebachiensis* persistence is low in outer areas, and relatively high at intermediate gradient values in inner areas. The mixed GAM analysis of the Temperature Atlas data showed that the coldest waters occur at intermediate positions along the coast-ocean gradient, indicating a relationship between temperature and sea urchin occurrence along this gradient. The increased probability of sea urchins at these intermediate positions could also be related to nutrient supply. Sea urchins in the vicinity of kelp forests may receive sufficient nourishment through supply of drift algae. The importance of drift algae to sea urchins are well documented by [56].

The influence of depth is likely associated to its influence on light and other factors such as reduced water movement related to waves. Our results imply that *Laminaria hyperborea* mainly recover in shallow, wave exposed, high saline and outer areas, i.e. areas that enhance growth through clear water and high water movement for exchange of dissolved nutrients and oxygen across the diffusive boundary layer [57], [58]. The two main interactions in the BRT-model for kelp imply increased probability for kelp recovery in shallow, hilly, wave exposed areas, with good conditions for kelp growth.

High curvature implies presence of appropriate substrate (hard bottom) for the kelp *Laminaria hyperborea* compared to the basins that have low curvature. Hence the influence of curvature on kelp is most likely a proxy for rocky bottom. The best *Strongylocentrotus droebachiensis* GAM model did not include curvature and slope and these predictors had a low importance in the BRT-model. This implies that substrate type (slope as well as curvature is a proxy for substrate), are of less importance for sea urchins than for kelp. *S. droebachiensis* is found in high densities at both rocky and sandy sea beds (observations during these field studies).

The probability of sea urchin occurrence increased northward, whereas recovery of kelp had a more variable response to latitude. How these responses result in regional differences due to local variation in topography and physical factors such as wave exposure and salinity, was delineated by dividing the study area into 5 equally sized regions. This subdivision shows that there is a marked difference in the relative distribution of the two states between the two northernmost (region 4 and 5) and the three southern regions (region 1, 2 and 3). Based on the BRT analysis the sea urchins seemed to prevail in 30–70% of the shallow areas in the northernmost regions, whereas they prevailed in only 0.1–4% of the areas in the southern regions. Recovery of kelp showed a more varied pattern with maximum recovery in region 2 (75% recovery) and about 55% in region 1 and 3, and only 38 and 19% in region 4 and 5 respectively. This indicates a recovery border between south (region 1–3) and north (region 4–5), but also show that the regional differences in other influential factors, such as wave exposure, salinity and curvature, may cause a more rapid recovery of kelp in certain areas/regions (as showed for region 2). A similar border between outer and inner areas was indicated by the BRT analysis. Both borders could be related to temperature above some threshold value that affect sea urchins negatively, e.g. in the spring period (affecting the newly released larvae) or in the summer (affecting settlement of sea urchin larvae, or e.g. reducing growth of the adult sea urchins). However the proximity of outer areas to intact kelp forests is likely to have a positive influence on kelp recovery in the outer areas through supply of spores, and to sea urchin persistence in the intermediate inner area due to supply of drift algae.

The GAM analysis indicated that high current speed rates were less favorable for sea urchins, but that the sea urchins benefited of having at least some current speed. Moreover, the BRT model of *Strongylocentrotus droebachiensis* persistence showed higher probability of sea urchin persistence in areas with speed velocities of about 0.2 m/s. This matches the current speed values found to be optimal for *S. droebachiensis* foraging by [59]. This signal of a positive influence of this optimal current speed level was found along the entire latitudinal gradient in the study area. Hence, current speed may be more important to explain the distribution of sea urchins and kelp than previously assumed. This underlines the importance of investigations of climate change on wave and current regimes, and how changes in ocean climate might influence kelp and sea urchins.

What further development of kelp recovery is indicated by the results of this study? Within the southern, recovered area there are small patches with pebbles and fissured rocky substrate that inhabit small sea urchins (personal observations) that may be able to respond to improved conditions for sea urchin recruitment. Cryptic behavior in young *Strongylocentrotus droebachiensis* between stones is shown by [60]. [61] and [62] show that refuge sea urchin populations are capable of a rapid comeback to areas that are not recovered by kelp. According to the BRT-models, 20–40% of the study area is likely to lack *Laminaria hyperborea* and *S*. *droebachiensis*. Hence a relatively large area seems to be vulnerable for an increase in sea urchin density. This instability, combined with too high temperatures for kelp in near future would give bleak prospects for kelp recovery.

The results indicate a relationship between reduced sea urchin densities, kelp recovery and climate change. A persistent trend of warm spring and summer temperatures could be a prerequisite for the low abundance of sea urchins in the three southernmost regions. For the time being, temperature increase in the study area are considerably below the threshold temperature (15°C) found by [63] to inhibit new frond formation for *Laminaria hyperborea*, and well below critical limits for harmful influence on *L. hyperborea* gametophytes (21°C, according to [64]). Hence, an increase in temperature of maximum 2 degrees, as forecasted by [65] for the next decade, is within the range of a positive influence on kelp growth and survival. This is in contrast to the expected decrease of *L. hyperborea* and three other kelp species presence and abundance in UK and Ireland [66], as well as the forecasted decrease of *L. digitata* within Western Europe as a response to warmer climate [67]. Further, kelp forest is considered as a stable state [2], [4], [9] that are likely to inhabit sea urchin predators that may prevent overgrazing [2] even if future temperature should drop. Moreover, [2] suggest several mechanisms that reinforce a flip back to a stable kelp forest community when macroalgae start to recover. These mechanisms include macroalgae housing predators to juvenile sea urchins and being a nursery habitat for crabs, resulting in increased predation of adult sea urchins from an increased crab population. According to analysis of a size structured kelp population dynamic model, recovery of kelp in areas with good growth conditions, providing large canopy plants, also imply a stabilizing influence on kelp populations dynamics [68] and increased resilience to overgrazing in areas experiencing kelp recovery.

This study has revealed a number of physical factors that influence the recovery of kelp and the spatial distribution of kelps and sea urchins. Yet, a comprehensive understanding of the ongoing changes includes both direct and indirect effects of these factors also on biological interactions such as competition, grazing and predation. Elevated water temperatures lead to northwards migration of organisms that may influence the dynamic between kelp and sea urchins. Many species in the North Atlantic has extended their distribution northward during the last decades (e.g. [69], [70]). [19] documents predation on sea urchins by the edible crab (*Cancer pagurus*), and the abundance of crab has increased tremendously within the kelp recovery area during recent decades [71]. Increased predation pressure on sea urchins may add to drive the observed kelp recovery in the south. Great influence of predators on sea urchin densities in NW Atlantic is shown by [2].

The BRT models explained a high degree of the variation in kelp recovery and persistence of sea urchins, and had an excellent fit when applied to the test data (AUC-values ≥0.9), hence the predictions from the models are likely to give an appropriate picture of the 2004–2011 distribution of the two species. The recovery of kelp is of great importance for the coastal ecosystems biodiversity, functions and services, as kelp forests have high primary and secondary production, and house a high number of species and individuals [66], [72]–[75]. The recovery of about 50% of previously barren areas are likely to have a positive influence on coastal fisheries and coastal communities that are closely related to marine resources [74].

## Supporting Information

Figure S1 **The BRT model for kelp recovery.** The partial response plots of the BRT model for recovery of the kelp *Laminaria hyperborea*. Relative importance of each factor is included in brackets.
(TIF)

Figure S2 **Calibration plots for the kelp models.** Calibration plots for; the best GAM, the average GAM with and without interactions, and for the BRT model of recovery of the kelp *L. hyperborea*, when applied to test data. There is a close relationship between the observed occurrences as proportion of surveyed sites versus predicted probability across the range of probability classes. Number of observations per probability class is shown above each bar.
(TIFF)

Figure S3 **The BRT model for sea urchin persistence.** The partial response plots of the BRT model for presence/persistence of the sea urchin *Strongylocentrotus droebachiensis*. Relative importance of each factor is included in brackets.
(TIFF)

Figure S4 **Calibration plots for the sea urchin models.** Calibration plots for; the best GAM with and without interaction, the average GAM with and without interactions, and for the BRT model, for presence/persistence of the sea urchin *Strongylocentrotus droebachiensis*, when applied to test data. There is a close relationship between the observed occurrences as proportion of surveyed sites versus predicted probability across the range of probability classes. Number of observations per probability class is shown above each bar.
(TIFF)

Figure S5 **Temperature Atlas analysis.** Partial response plots of the mixed GAM for sea water temperature at 10 m depth for the period 1990–2007, as a function of latitude, coast-ocean gradient (i.e. the residuals for the linear relationship between latitude and longitude), year and seasons (represented by the mid-month in each season). The analysis is based on data from the Temperature Atlas developed by [28].
(TIFF)

Table S1 **The environmental predictors.** Description of the environmental factors that were used in the statistical analyses of kelp (*Laminaria hyperborea*) and sea urchin (*Strongylocentrotus droebachiensis*) distribution.
(DOCX)

Table S2 **GAMs for kelp recovery.** Overview of the 8 best GAMs (one column per model, increasing AICc values to the right) for kelp *Laminaria hyperborea* recovery (i.e. delta AICc<4). Factors included in each model is marked with +. Parameters included are the models degrees of freedom (df), Loglikelihood value, AICc, ΔAICc and weight.
(DOCX)

Table S3 **GAMs for sea urchin persistence.** Overview of the 36 best GAMs models (one column per model, increasing AICc values to the right; the first 18 models on top, the last 18 models below) for sea urchin *Strongylocentrotus droebachiensis* persistence (i.e. delta AICc-values less than 4). Factors included in each model is marked with +. Parameters included are the models degrees of freedom (df), Loglikelihood value (LogLik), AICc-value, delta AICc-value and weight.
(DOCX)
