# Supplementary figures and images for: The Influence of Physical Factors on Kelp and Sea Urchin Distribution in Previously and Still Grazed Areas in the NE Atlantic
Source: PLoS One. 2014 Jun 20;9(6):e100222. doi: 10.1371/journal.pone.0100222 (PMC4064999; doi:10.1371/journal.pone.0100222)

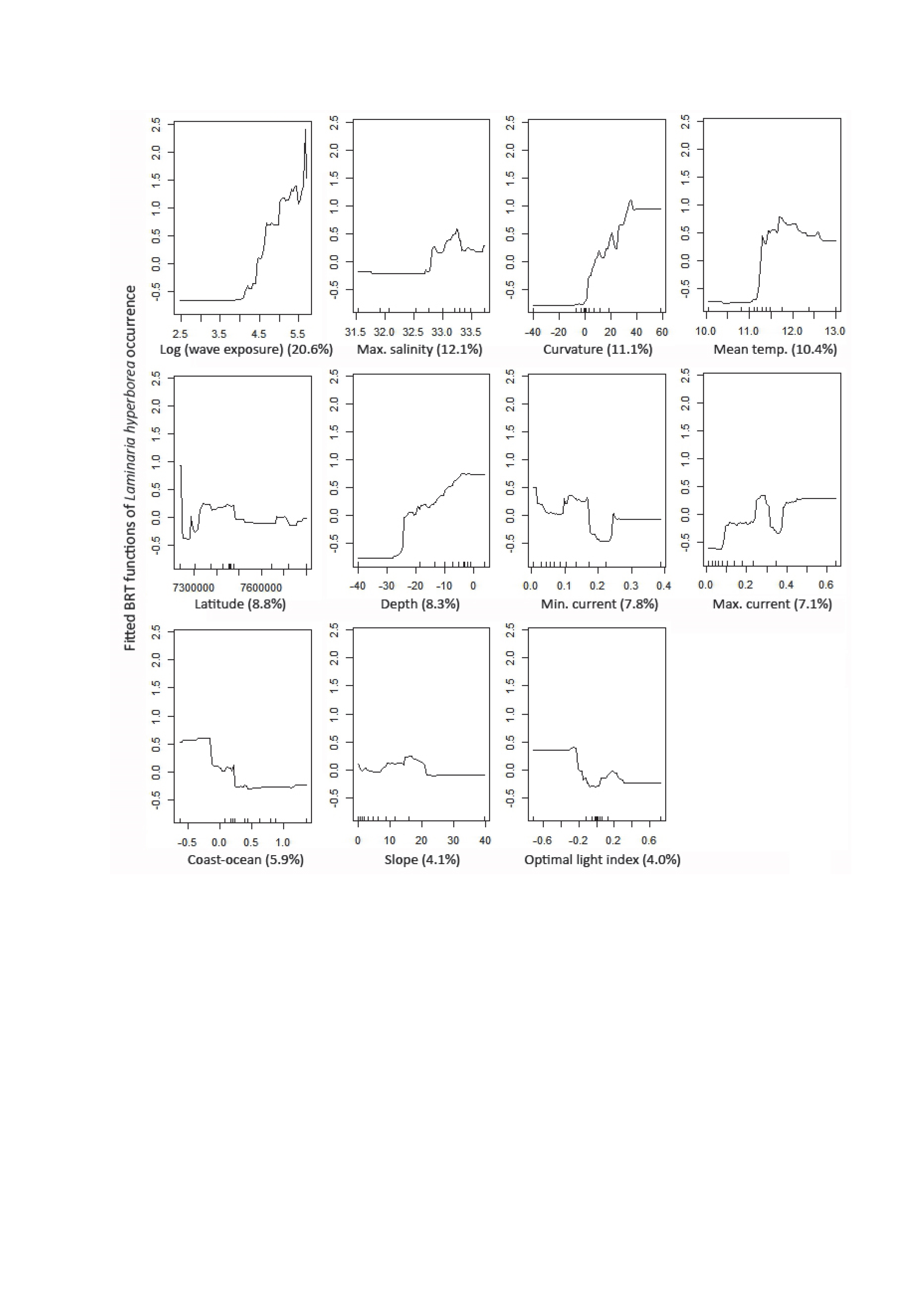

Supplement: Figure S1 — The BRT model for kelp recovery. The partial response plots of the BRT model for recovery of the kelp Laminaria hyperborea. Relative importance of each factor is included in brackets. (TIF) [file pone.0100222.s001.tif]

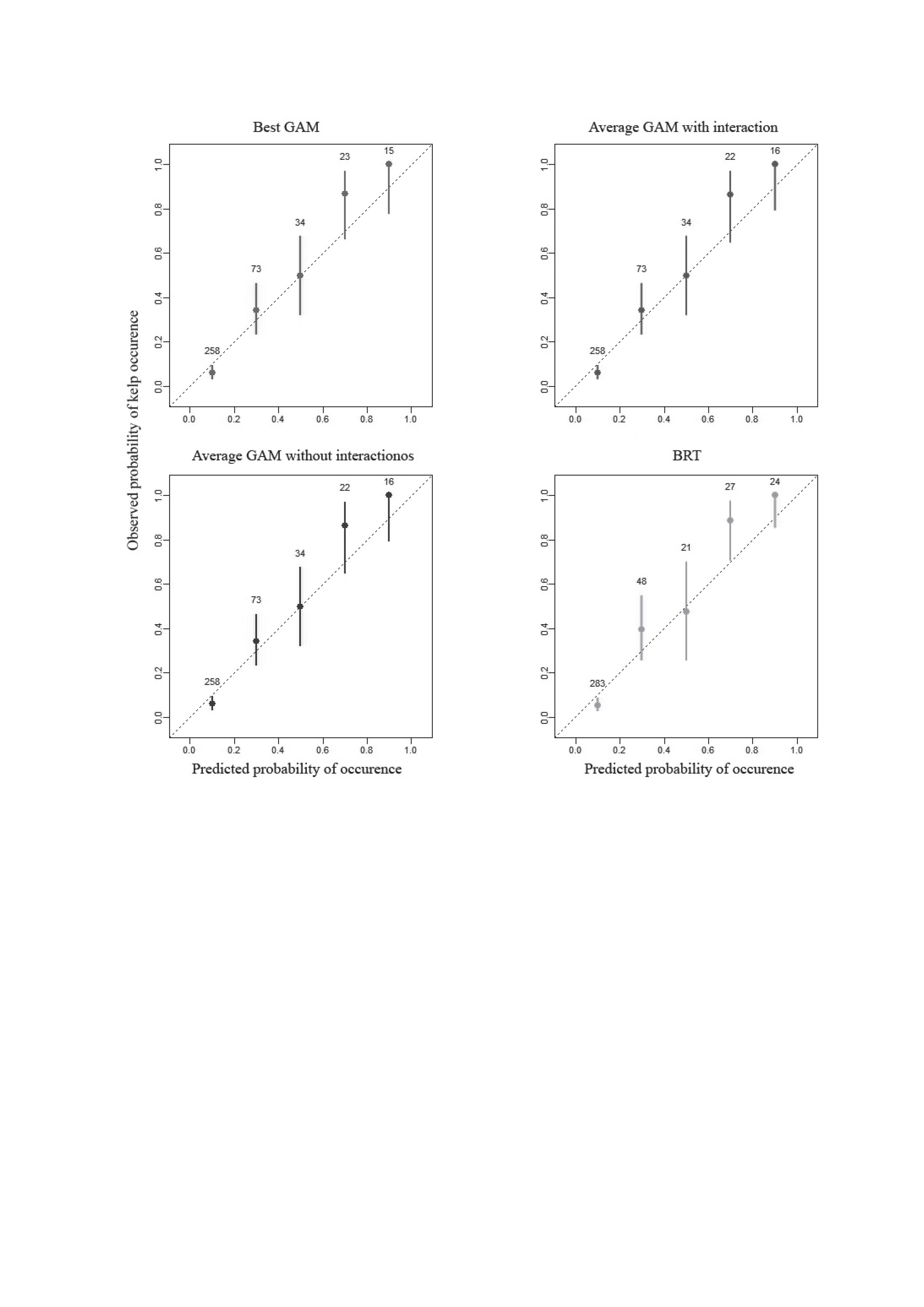

Supplement: Figure S2 — Calibration plots for the kelp models. Calibration plots for; the best GAM, the average GAM with and without interactions, and for the BRT model of recovery of the kelp L. hyperborea, when applied to test data. There is a close relationship between the observed occurrences as proportion of surveyed sites versus predicted probability across the range of probability classes. Number of observations per probability class is shown above each bar. (TIFF) [file pone.0100222.s002.tif]

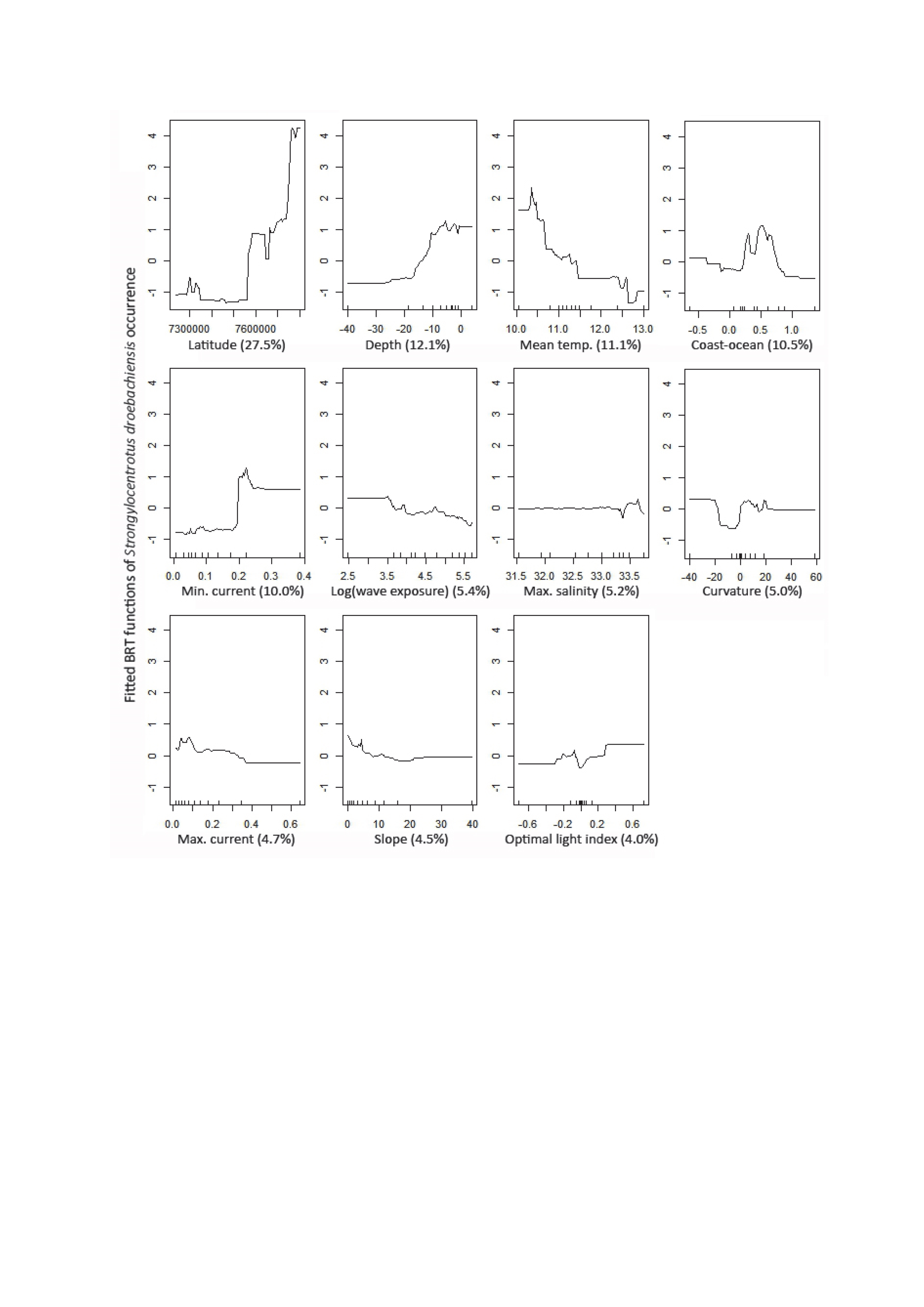

Supplement: Figure S3 — The BRT model for sea urchin persistence. The partial response plots of the BRT model for presence/persistence of the sea urchin Strongylocentrotus droebachiensis. Relative importance of each factor is included in brackets. (TIFF) [file pone.0100222.s003.tif]

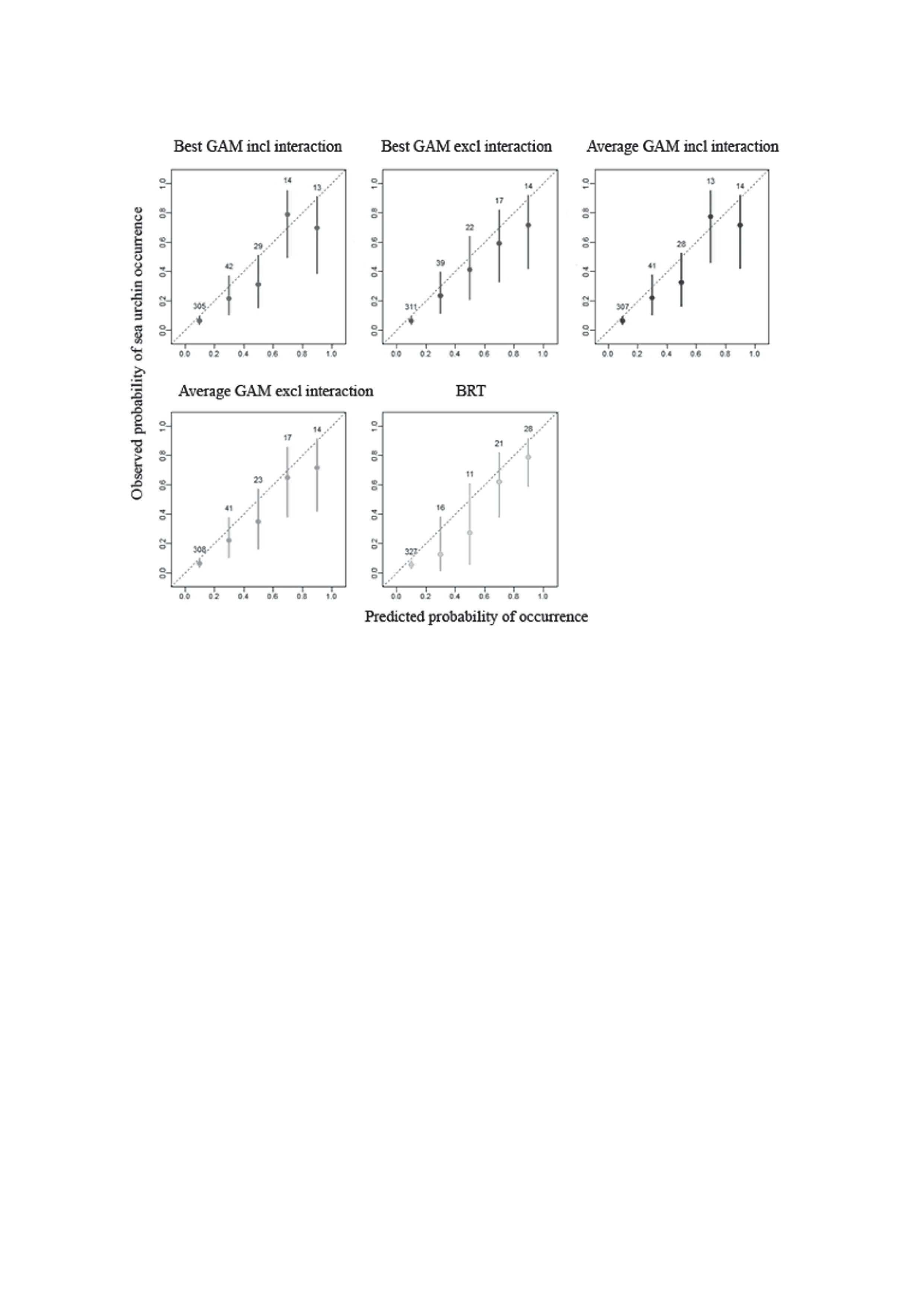

Supplement: Figure S4 — Calibration plots for the sea urchin models. Calibration plots for; the best GAM with and without interaction, the average GAM with and without interactions, and for the BRT model, for presence/persistence of the sea urchin Strongylocentrotus droebachiensis, when applied to test data. There is a close relationship between the observed occurrences as proportion of surveyed sites versus predicted probability across the range of probability classes. Number of observations per probability class is shown above each bar. (TIFF) [file pone.0100222.s004.tif]

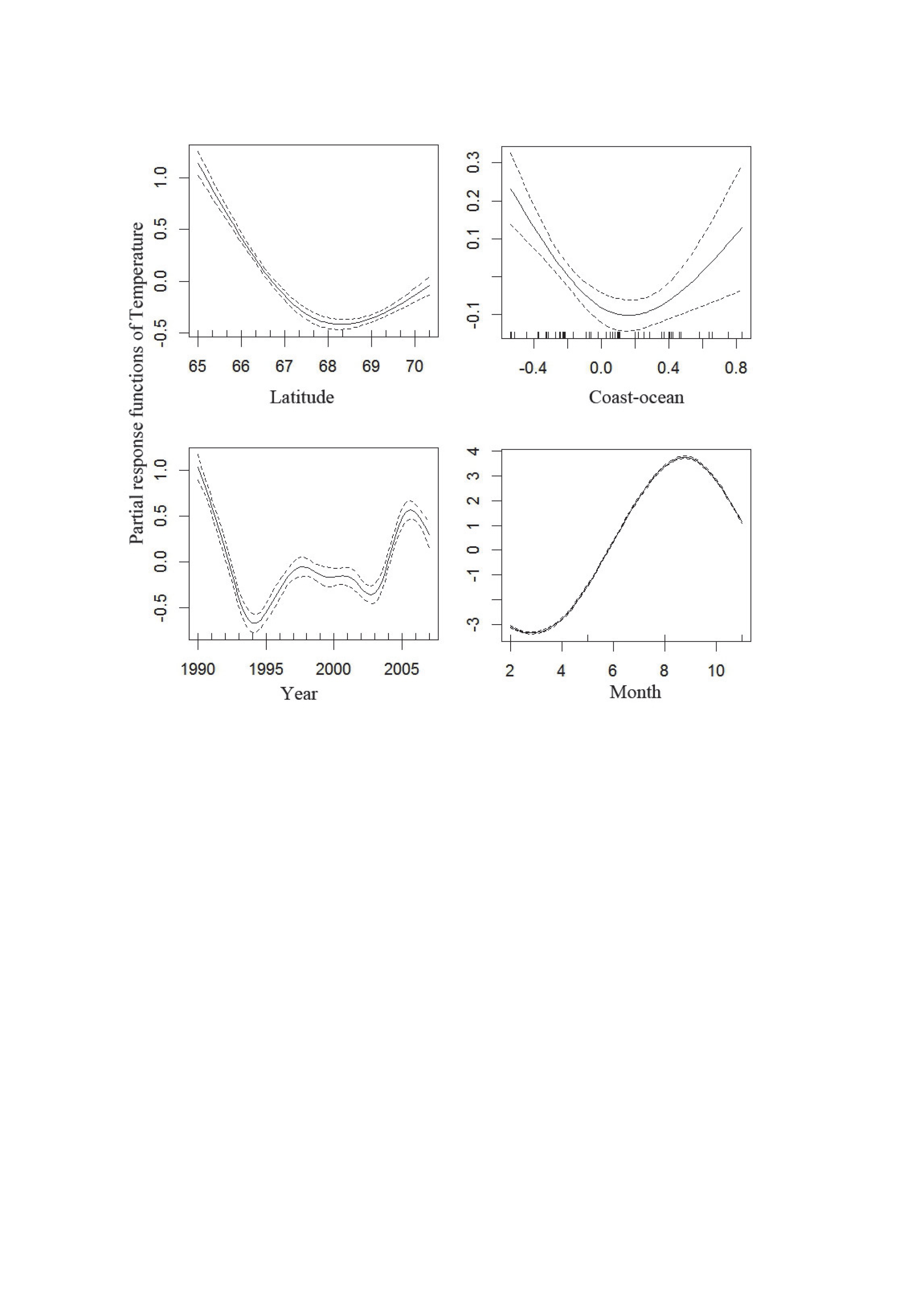

Supplement: Figure S5 — Temperature Atlas analysis. Partial response plots of the mixed GAM for sea water temperature at 10 m depth for the period 1990–2007, as a function of latitude, coast-ocean gradient (i.e. the residuals for the linear relationship between latitude and longitude), year and seasons (represented by the mid-month in each season). The analysis is based on data from the Temperature Atlas developed by [28]. (TIFF) [file pone.0100222.s005.tif]
