# Supplementary material for: The Influence of Physical Factors on Kelp and Sea Urchin Distribution in Previously and Still Grazed Areas in the NE Atlantic
Source: PLoS One. 2014 Jun 20;9(6):e100222. doi: 10.1371/journal.pone.0100222 (PMC4064999; doi:10.1371/journal.pone.0100222)
Supplement: Table S1 — The environmental predictors. Description of the environmental factors that were used in the statistical analyses of kelp (Laminaria hyperborea) and sea urchin (Strongylocentrotus droebachiensis) distribution. (DOCX) [file pone.0100222.s006.docx]

**Table S1**. Description of the environmental factors that were used in the statistical analyses and distribution modeling of kelp (*Laminaria hyperborea*) and sea urchin (*Strongylocentrotus droebachiensis*).

| **Depth**; Measured in field with underwater camera with depth sensor. The geographic position was determined with a Garmin GPS, accuracy: ± 5 m. For spatial predictive modeling we used a digital terrain model (DTM) developed in the Norwegian National program for mapping of marine biodiversity (Bekkby et al. 2013). |
| --- |
| **Slope**; Calculated as maximum change from each DTM cell to its eight closest neighbors (in degrees) using the ArcGIS 10.0 “Slope” function. The slope range within the study area was 0-88.1 degrees. |
| **Terrain curvature**; Calculated in ArcGIS 10.0 as the difference between the depth in each DTM grid cell and the mean depth in a moving neighborhood, defined by the size of the calculation window (similar to the bathymetric position index, described by Wilson et al. 2007). We used a 525 m calculation window. A negative grid cell value indicates that the grid cell is within a basin; a positive value indicates a shoal. The more negative the value, the deeper the basin; the more positive the value, the greater the rise in the shoal. |
| **Optimal light index**; This index depends on bottom slope and aspect, and is estimated as the deviance of influx of light in a grid cell compared to the optimal levels at the assumed optimal slope (45°) and aspect (202.5°). The index was developed for terrestrial vegetation (Parker 1988), and has been further developed by Økland (1990, 1996). The index was included in the best GAM kelp model in an earlier study (Bekkby et al. 2009). The slope and aspect is calculated from the DTM, and the index is calculated by using an ArcView 3.3 script. The index ranges from -7.5 to 20.2 and is positive at aspects 202.5° ± 90° (regardless of slope) and negative for aspect values (202.5° - 180°) ± 90°. |
| **Coast-ocean gradient**; This southeast-northwest gradient replace longitude as a coast-ocean gradient because longitude is strongly correlated to latitude (r^2^ = 0.93) due to the northeast orientation of the coastline. The gradient is estimated as the standardized residuals from the linear relationship between longitude and latitude. These residuals are not correlated to latitude (ρ = 0.30). |
| **Latitude;** geographic position along the north-south gradient. |
| **Wave exposure**; A wave exposure index is modeled with a spatial resolution of 25 m, using data on fetch (distance to nearest shore, island, or coast) and wind strength in 16 directions (delivered by the Norwegian Meteorological Institute), averaged over a 10-year period (i.e. 1995-2004). The model (Isæus 2004) has been developed for the whole Norwegian coast and has been applied in predictive distribution models of e.g. kelp (*Laminaria hyperborea*, Bekkby et al. 2009, Norderhaug & Christie 2009) and eelgrass (*Zostera marina*, Bekkby et al. 2008) at the West coast of Norway. The wave exposure values of the study area range from sheltered to very exposed (cf Wijkmark & Isæus 2010, similar to the EUNIS system of Davies & Moss 2004). |
| **Current speed, salinity and temperature;** A three-dimensional hydrodynamical model (ROMS, Haidvogel et al. 2008) with a spatial resolution of 800 m (NorKyst, Albretsen et al. 2011) provided monthly averages from 5 m depth for August 2009. ROMS has shown good results when compared with field observations (LaCasce et al. 2007) and has users worldwide (myroms.org). Available predictors from the model include current speed, temperature (at 10 m depth) and salinity (mean, minimum, maximum and standard deviation). The current fields are calculated in eastward and northward components. Hence, the resulting speed is the combination of the speed in each component, respectively, and the associated direction. We wanted to use uncorrelated predictors and chose to use *temperature mean values*, *minimum* and *maximum* *current speed*, and *salinity maximum values*. The salinity maximum value was chosen as it came out to be the most important of the salinity predictors in the full BRT models containing all possible salinity predictors. |

**Table S1 References**

Albretsen J, Sperrevik AK, Staalstrøm A, Sandvik AD, Vikebø F, Asplin L (2011) NorKyst800 Report No.1 User Manual and technical descriptions, Fisken og havet nr.2/2011.

Bekkby T, Rinde E, Erikstad L, Bakkestuen V, Longva O, et al. (2008) Spatial probability modelling of eelgrass (*Zostera marina*) distribution on the west coast of Norway. ICES J Mar Sci 65: 1093-1101.

Bekkby T, Rinde E, Erikstad L, Bakkestuen V (2009) Spatial predictive distribution modelling of the kelp species *Laminaria hyperborea*. ICES J Mar Sci 66: 2106-2115

Bekkby T, Moy FE, Olsen H, Rinde E, Bodvin T, et al. (2013) The Norwegian Program for Mapping of Marine Habitats - Providing Knowledge and Maps for ICZMP. Chapter 2, page 21-30 in: Moksness E, Dahl E and Støttrup J (Eds.) Global Challenges in Integrated Coastal Zone Management, Vol II. John Wiley & Sons, Ltd, Oxford, UK.

Davies CE, Moss D, Hill MO (2004) EUNIS Habitat Classification, Revised 2004. Report to European Environment Agency, European Topic Centre on Nature Protection and Biodiversity. October 2004.

Haidvogel D, Arango H, Budgell W, Cornuelle B, Curchitser E, et al. (2008) Ocean forecasting in terrain-following coordinates: Formulation and skill assessment of the Regional Ocean Modeling System. Jour Comp Phys 227: 3595-3624.

Isæus M (2004) Factors structuring *Fucus* communities at open and complex coastlines in the Baltic Sea. Filosofie doktorexamen, Stockholms universitet, Stockholm.

LaCasce JH, Røed LP, Bertino L, Ådlandsvik B (2007) CONMAN Technical Report No. 2: Analysis of model results, met.no Report 5/2007.

Norderhaug KM, Christie HC (2009) Sea urchin grazing and kelp re-vegetation in the NE Atlantic. Mar Biol Res 5: 515-528

Parker KC (1988) Environmental relationships and vegetation associates of columnar cacti in the northern Sonoran desert. Vegetation, 78: 125-140.

Wijkmark N, Isæus M (2010) Wave exposure calculations for the Baltic Sea, AquaBiota Report 2010:02.

Wilson MFJ, O’Connell B, Brown C, Guinan JC, Grehan AJ (2007) Multiscale Terrain Analysis of Multibeam Bathymetry Data for Habitat Mapping on the Continental Slope. Marine Geodesy 30: 3-35.

Økland T (1990) Vegetational and ecological monitoring of boreal forests in Norway. 1. Rausjømarka in Akershus county, SE Norway. Sommerfeltia 10: 1–52.

Økland T (1996) Vegetation–environment relationships of boreal spruce forests in ten monitoring reference areas in Norway. Sommerfeltia 22: 1–349.
