# Supplementary material for: The Influence of Physical Factors on Kelp and Sea Urchin Distribution in Previously and Still Grazed Areas in the NE Atlantic
Source: PLoS One. 2014 Jun 20;9(6):e100222. doi: 10.1371/journal.pone.0100222 (PMC4064999; doi:10.1371/journal.pone.0100222)
Supplement: Table S2 — GAMs for kelp recovery. Overview of the 8 best GAMs (one column per model, increasing AICc values to the right) for kelp Laminaria hyperborea recovery (i.e. delta AICc<4). Factors included in each model is marked with +. Parameters included are the models degrees of freedom (df), Loglikelihood value, AICc, ΔAICc and weight. (DOCX) [file pone.0100222.s007.docx]

**Table S2**. Overview of the 8 best GAM models (one column per model, increasing AICc values to the right) for kelp *Laminaria hyperborea* recovery (i.e. ∆AICc<4). Predictors included in each model is marked with +. Parameters included are the models degrees of freedom (df), Loglikelihood value, AICc, ∆AICc and weight.

| **Mod nr** | **2000** | **3983** | **2016** | **4015** | **2032** | **4047** | **4079** | **2048** |
| --- | --- | --- | --- | --- | --- | --- | --- | --- |
| Intercept | -2.5 | -2.5 | -2.5 | -2.5 | -2.5 | -2.5 | -2.5 | -2.5 |
| Depth | + |  | + |  | + |  |  | + |
| Depth x log(wave exposure) |  | + |  | + |  | + | + |  |
| Terrain curvature | + | + | + | + | + | + | + | + |
| Latitude | + | + | + | + | + | + | + | + |
| Log (wave exposure) | + |  | + |  | + |  |  | + |
| Coast-ocean gradient |  |  | + | + |  |  | + | + |
| Optimal light index |  |  |  |  | + | + | + | + |
| Max. salinity | + | + | + | + | + | + | + | + |
| Slope | + | + | + | + | + | + | + | + |
| Max. current speed | + | + | + | + | + | + | + | + |
| Min. current speed | + | + | + | + | + | + | + | + |
| Mean temperature | + | + | + | + | + | + | + | + |
| *Df* | *14.45* | *14.45* | *15.51* | *15.51* | *15.38* | *15.38* | *16.45* | *16.45* |
| *Loglikelihood* | *-379.85* | *-379.85* | *-379.60* | *-379.60* | *-379.85* | *-379.85* | *-379.60* | *-379.60* |
| *AICc* | *788.96* | *788.96* | *790.64* | *790.64* | *790.88* | *790.88* | *792.59* | *792.59* |
| *∆AICc* | *0.00* | *0.00* | *1.68* | *1.68* | *1.92* | *1.92* | *3.63* | *3.63* |
| *Weight* | *0.25* | *0.25* | *0.11* | *0.11* | *0.10* | *0.10* | *0.04* | *0.04* |
|  |  |  |  |  |  |  |  |  |
