# Supplementary material for: The Influence of Physical Factors on Kelp and Sea Urchin Distribution in Previously and Still Grazed Areas in the NE Atlantic
Source: PLoS One. 2014 Jun 20;9(6):e100222. doi: 10.1371/journal.pone.0100222 (PMC4064999; doi:10.1371/journal.pone.0100222)
Supplement: Table S3 — GAMs for sea urchin persistence. Overview of the 36 best GAMs models (one column per model, increasing AICc values to the right; the first 18 models on top, the last 18 models below) for sea urchin Strongylocentrotus droebachiensis persistence (i.e. delta AICc-values less than 4). Factors included in each model is marked with +. Parameters included are the models degrees of freedom (df), Loglikelihood value (LogLik), AICc-value, delta AICc-value and weight. (DOCX) [file pone.0100222.s008.docx]

**Table S3**. Overview of the 36 best GAM models (one column per model, increasing AICc values to the right; the first 18 models on top, the last 18 models below) for sea urchin *Strongylocentrotus droebachiensis* persistence (i.e. ∆AICc-values less than 4). Predictors included in each model is marked with +. Parameters included are the models degrees of freedom (df), Loglikelihood value (LogLik), AICc-value, ∆AICc-value and weight.

The first 18 models:

| **Mod nr:** | **3755** | **3627** | **3691** | **4011** | **3819** | **3759** | **4075** | **3947** | **4015** | **3243** | **3883** | **1822** | **3706** | **3499** | **3631** | **3695** | **3823** | **4079** |
| --- | --- | --- | --- | --- | --- | --- | --- | --- | --- | --- | --- | --- | --- | --- | --- | --- | --- | --- |
| Intercept | -2.28 | -2.25 | -2.27 | -2.29 | -2.29 | -2.29 | -2.30 | -2.27 | -2.30 | -2.27 | -2.26 | -2.27 | -2.28 | -2.28 | -2.26 | -2.27 | -2.29 | -2.30 |
| Depth |  |  |  |  |  |  |  |  |  |  |  | + | + |  |  |  |  |  |
| Depth x log(wave exposure) | + | + | + | + | + | + | + | + | + | + | + |  |  | + | + | + | + | + |
| Terrain curvature |  |  |  |  |  | + |  |  | + |  |  |  |  |  | + | + | + | + |
| Latitude | + | + | + | + | + | + | + | + | + | + | + | + | + | + | + | + | + | + |
| Log (wave exposure) |  |  |  |  |  |  |  |  |  |  |  | + | + |  |  |  |  |  |
| Coast-ocean gradient | + | + | + | + | + | + | + | + | + | + | + | + | + | + | + | + | + | + |
| Optimal light index |  |  | + |  | + |  | + | + |  |  |  |  | + |  |  | + | + | + |
| Max. salinity | + |  |  | + | + | + | + |  | + | + |  |  |  | + |  |  | + | + |
| Slope |  |  |  | + |  |  | + | + | + |  | + |  |  | + |  |  |  | + |
| Max. current speed | + | + | + | + | + | + | + | + | + |  | + | + | + |  | + | + | + | + |
| Min. current speed | + | + | + | + | + | + | + | + | + | + | + | + | + | + | + | + | + | + |
| Mean temperature | + | + | + | + | + | + | + | + | + | + | + | + | + | + | + | + | + | + |
| *df* | *15.30* | *13.59* | *14.54* | *16.41* | *16.17* | *16.17* | *17.32* | *15.61* | *17.29* | *14.40* | *14.65* | *13.05* | *14.06* | *15.50* | *14.44* | *15.40* | *17.07* | *18.22* |
| *LogLik* | *-372.50* | *-374.43* | *-373.52* | *-371.69* | *-372.00* | *-372.22* | *-371.06* | *-372.88* | *-371.16* | *-374.13* | *-373.90* | *-375.59* | *-374.60* | *-373.15* | *-374.34* | *-373.48* | *-371.79* | *370.62* |
| *AICc* | *776.01* | *776.37* | *776.49* | *776.68* | *776.81* | *777.25* | *777.28* | *777.40* | *777.42* | *777.43* | *777.49* | *777.60* | *777.66* | *777.73* | *777.93* | *778.18* | *778.23* | *778.26* |
| *∆AICc* | *0.00* | *0.36* | *0.48* | *0.67* | *0.80* | *1.24* | *1.28* | *1.40* | *1.41* | *1.42* | *1.48* | *1.59* | *1.65* | *1.72* | *1.92* | *2.17* | *2.22* | *2.25* |
| *Weight* | *0.07* | *0.06* | *0.05* | *0.05* | *0.05* | *0.04* | *0.04* | *0.03* | *0.03* | *0.03* | *0.03* | *0.03* | *0.03* | *0.03* | *0.03* | *0.02* | *0.02* | *0.02* |
|  |  |  |  |  |  |  |  |  |  |  |  |  |  |  |  |  |  |  |

| **Table S3** continues. The last 18 models: | | | | | | | | | | | | | |  | |  | |  | |  | |  | |  | |  | |  | |  | |  | |  | |  | |
| --- | --- | --- | --- | --- | --- | --- | --- | --- | --- | --- | --- | --- | --- | --- | --- | --- | --- | --- | --- | --- | --- | --- | --- | --- | --- | --- | --- | --- | --- | --- | --- | --- | --- | --- | --- | --- | --- |
| **Mod nr:** | | **3307** | | **3563** | | **4026** | | **3887** | | **3951** | | **3503** | | **3247** | | **3962** | | **4090** | | **3115** | | **3898** | | **3646** | | **3179** | | **3710** | | **3567** | | **3770** | | **3435** | | **3371** | |
| Intercept | | -2.28 | | -2.29 | | -2.31 | | -2.26 | | -2.27 | | -2.29 | | -2.28 | | -2.28 | | -2.31 | | -2.23 | | -2.27 | | -2.27 | | -2.24 | | -2.28 | | -2.29 | | -2.29 | | -2.25 | | -2.23 | |
| Depth | |  | |  | | + | |  | |  | |  | |  | | + | | + | |  | | + | | + | |  | | + | |  | | + | |  | |  | |
| Depth x log(wave exposure) | | + | | + | |  | | + | | + | | + | | + | |  | |  | | + | |  | |  | | + | |  | | + | |  | | + | | + | |
| Terrain curvature | |  | |  | |  | | + | | + | | + | | + | |  | |  | |  | |  | | + | |  | | + | | + | |  | |  | |  | |
| Latitude | | + | | + | | + | | + | | + | | + | | + | | + | | + | | + | | + | | + | | + | | + | | + | | + | | + | | + | |
| Log (wave exposure) | |  | |  | | + | |  | |  | |  | |  | | + | | + | |  | | + | | + | |  | | + | |  | | + | |  | |  | |
| Coast-ocean gradient | | + | | + | | + | | + | | + | | + | | + | | + | | + | | + | | + | | + | | + | | + | | + | | + | | + | | + | |
| Optimal light index | | + | | + | |  | |  | | + | |  | |  | | + | | + | |  | |  | |  | | + | | + | | + | |  | | + | |  | |
| Max. salinity | | + | | + | | + | |  | |  | | + | | + | |  | | + | |  | |  | |  | |  | |  | | + | | + | |  | |  | |
| Slope | |  | | + | | + | | + | | + | | + | |  | | + | | + | |  | | + | |  | |  | |  | | + | |  | | + | | + | |
| Max. current speed | |  | |  | | + | | + | | + | |  | |  | | + | | + | |  | | + | | + | |  | | + | |  | | + | |  | |  | |
| Min. current speed | | + | | + | | + | | + | | + | | + | | + | | + | | + | | + | | + | | + | | + | | + | | + | | + | | + | | + | |
| Mean temperature | | + | | + | | + | | + | | + | | + | | + | | + | | + | | + | | + | | + | | + | | + | | + | | + | | + | | + | |
| *df* | | *15.32* | | *16.44* | | *15.81* | | *15.51* | | *16.48* | | *16.40* | | *15.31* | | *15.09* | | *16.75* | | *12.58* | | *14.09* | | *13.97* | | *13.53* | | *14.97* | | *17.36* | | *14.05* | | *14.62* | | *13.66* | |
| *LogLik* | | *373.62* | | *372.53* | | *373.24* | | *373.66* | | *372.70* | | *372.83* | | *374.02* | | *374.27* | | *372.59* | | *376.88* | | *375.35* | | *375.54* | | *375.99* | | *374.60* | | *372.28* | | *375.68* | | *375.17* | | *376.16* | |
| *AICc* | *778.31* | | *778.42* | | *778.55* | | *778.76* | | *778.84* | | *778.94* | | *779.08* | | *779.12* | | *779.16* | | *779.20* | | *779.23* | | *779.36* | | *779.37* | | *779.53* | | *779.82* | | *779.82* | | *779.96* | | *779.98* | |  |
| *AICc* | | *2.30* | | *2.41* | | *2.54* | | *2.75* | | *2.83* | | *2.93* | | *3.07* | | *3.11* | | *3.15* | | *3.19* | | *3.22* | | *3.35* | | *3.36* | | *3.52* | | *3.81* | | *3.81* | | *3.95* | | *3.97* | |
| *Weight* | | *0.02* | | *0.02* | | *0.02* | | *0.02* | | *0.02* | | *0.02* | | *0.01* | | *0.01* | | *0.01* | | *0.01* | | *0.01* | | *0.01* | | *0.01* | | *0.01* | | *0.01* | | *0.01* | | *0.01* | | *0.01* | |
